# Metals in Medicine

**DOI:** 10.1155/2012/705907

**Published:** 2012-08-23

**Authors:** Goran N. Kaluđerović, Santiago Gómez-Ruiz, Danijela Maksimović-Ivanić, Reinhard Paschke, Sanja Mijatović

**Affiliations:** ^1^Institut für Chemie, Martin-Luther-Universität Halle-Wittenberg, Kurt-Mothes-Straße 2, 06120 Halle, Germany; ^2^Departamento de Química Inorgánica y Analítica, ESCET, Universidad Rey Juan Carlos, 28933 Móstoles, Spain; ^3^Institute for Biological Research “Sinisa Stankovic,” University of Belgrade, Bulevar despota Stefana 142, 11060 Belgrade, Serbia; ^4^Biozentrum, Martin-Luther-Universität Halle-Wittenberg, Weinbergweg 22, 06120 Halle, Germany

Metals in medicine are bridging the areas of inorganic chemistry and medicine. Metal-based materials, metallodrugs, and agents for treating and detecting diseases, their synthesis, structure, and general properties, as well as biological applications on cellular and living system level, are of great importance. The mechanisms of action and the roles of these metal compounds in cellular regulation and signaling in health and diseases are of principal interest. These areas are linked by the need to involve researchers having a deep understanding of inorganic chemistry in medically relevant research. This special issue presents a collection of papers dealing with different compounds/materials investigated for antitumoral, antimicrobial, and antifungal activity as well as DNA binding study.

Y. Li et al. reported on the efficient and specific method for the determination of diphenyl-di-(2,4-difluobenzohydroxamato)tin(II), DPDFT, in rat plasma. Their preliminary studies indicated nonlinearity pharmacokinetics in the investigated dose ranges in rats and that the concentration-time curves of DPDFT in rat plasma could be fitted to two-compartment model. Additionally, results hinted that DPDFT might accumulate in certain organs, thus producing the toxicity, or could be quickly metabolized in the plasma into active antitumoral constituents.

The synthesis and characterization of novel salicylaldehyde-derived ligands and corresponding Cu(II), Co(II), Ni(II), and Zn(II) complexes are described by Kursunlu et al. Ligands bearing chlorine, bromine and –OH substituents showed moderate inhibition activity against some Gram-positive and Gram-negative bacteria including methicillin-resistant *Staphylococcus aureus*. Ni(II) and Zn(II) complexes were generally more effective against tested bacteria than Cu(II) and Co(II) complexes. 

In the work of A. A. Al-Amiery et al., significant antifungal activity of Cu(II), Co(II), and Ni(II) complexes with (*Z*)-2-(pyrrolidin-2-ylidene)hydrazinecarbothioamide and chloride ligands is described. The complexes were found to be superior antioxidants compared to ascorbic acid. 

Zietz et al. evaluated Cu release characteristics from Cu doped titanium alloy (Ti_6_Al_4_V) of antimicrobial implant surfaces *in vitro* according to the storage fluid and surface roughness. Plasma immersion ion implantation of Cu (Cu-PIII) and pulsed magnetron sputtering process of a titanium copper film (Ti-Cu) were applied to Ti_6_Al_4_V samples with different surface finishing of the implant material (polished, hydroxyapatite, and corundum blasted). The Cu concentration in the supernatant was measured using atomic absorption spectrometry.

M. Rezaee et al. investigated the optimum experimental conditions to prepare dry thin films of Pt compounds bound to plasmid DNA on a Ta substrate. Their results show that used conditions can induce damage to DNA and highly sensitize them to manipulations required to form thin films and recover DNA from the Ta substrate. The concentration of intact DNA increases significantly in the film samples when used lower incubation temperature and shorter incubation time. Thus, the optimum condition is obtained from equilibrium between temperature, time, and Pt-compounds concentration during the DNA platination reaction. 

In the review by S. Gómez-Ruiz et al., the mode of action of cisplatin against tumor cells as well as a brief outlook on the metallocene compounds as antitumor drugs and future tendencies for the use of the latter in anticancer chemotherapy are summarized. The authors reported on the molecular mechanisms of cisplatin interaction with DNA, DNA repair mechanisms, and cellular proteins. Molecular background of the sensitivity and resistance to cisplatin as well as its influence on the efficacy of the antitumor immune response were evaluated. Moreover, the use and mechanism of some metallocenes (titanocene, vanadocene, molybdocene, ferrocene and zirconocene) with high antitumor activity are reported. 

